# Supplementary material for: Polymorphisms in Pfkelch13 domains before and after the introduction of artemisinin-based combination therapy in Southwest Nigeria
Source: PLoS One. 2025 Mar 31;20(3):e0316479. doi: 10.1371/journal.pone.0316479 (PMC11957316; doi:10.1371/journal.pone.0316479)

Samples: 12912
Bases: 1095
Average spacing: 12

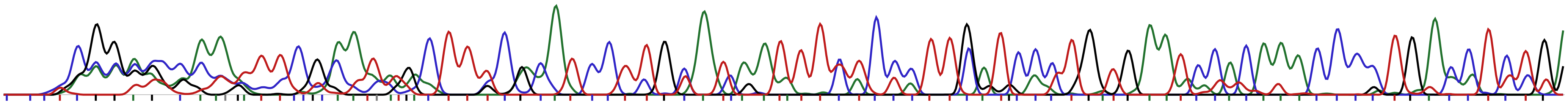

10 20 30 40 50 60 70 80 90

C C C T C G G A G C A A - G A T T C C G C A A T - A T G A C T T T C G C A T C C T T G C A T C A A T A T T C T C C C T T C A T G C C C T G A T G A A T C C A C A A A C C C C T G A C C T C T G

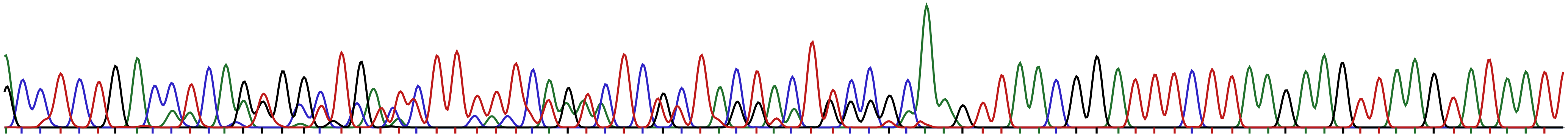

100 110 120 130 140 150 160 170

A C C T C T G A C C T C A G G G G C T G T T C T T T T T C A G T C T C G C T A C T A C T T C C G C A A G T T A A C G G A T T T C T T A A G A A G T T A A G T A T A A T

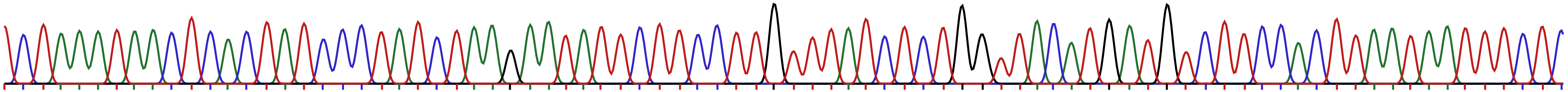

180 190 200 210 220 230 240 250 260

T C T A A A T A A C T C A C T A T C C C T A T C T A A G A A T A T T C T T C C T T G T T T A T C T C T G G T T A C A T G A T G T C T T C C A C T T A A T A A T T T C T C

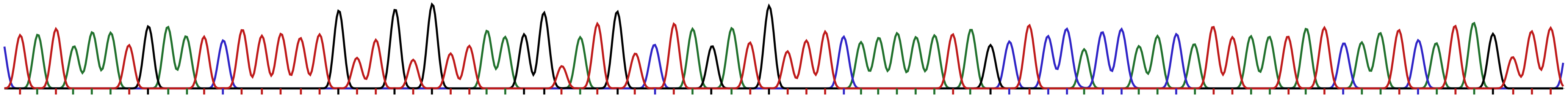

270 280 290 300 310 320 330 340

T A T A A A T G A A T C T T T T T G T T G T G T T A A G G T A T G T C T A G A T G T T T C A A A A A T A G C T C C A C C A A C A T T A A T A T C A A T C A T A G T T T

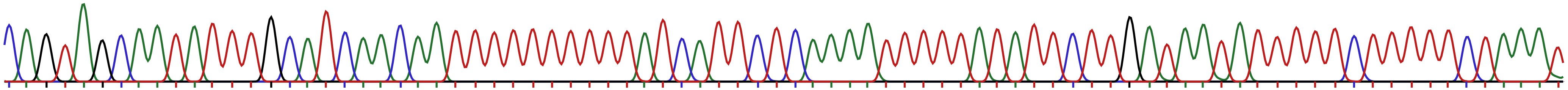

350 360 370 380 390 400 410 420

C A G T A G C A A T A T T T G C A T C A A C A A T T T T T T T T T T A T C A T T C T C A A A A T T T T T A T A T T C T T G A T A A T A T T T T T C T T T T T C T A A A T

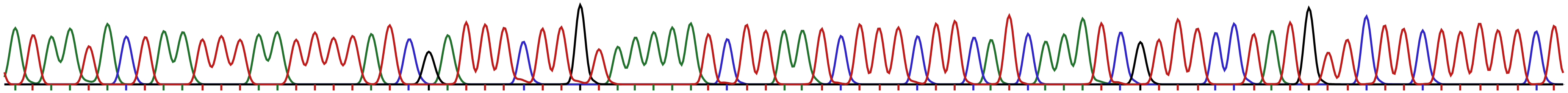

430 440 450 460 470 480 490 500 510

A T A A T A C T A A T T T A A T T T T A T C G A T T T C T T G T A A A A A T C T T A A T C T T T C T T C A T C A A A T C G T T T C C T A T G T T C T T C T T T T T C T

Samples: 12912
Bases: 1095
Average spacing: 12

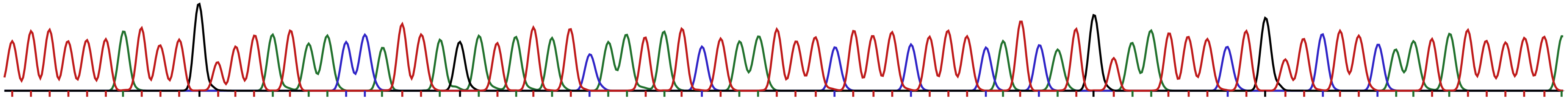

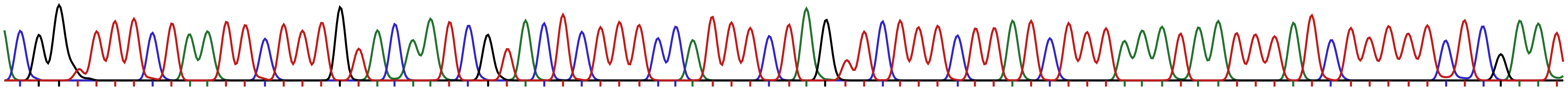

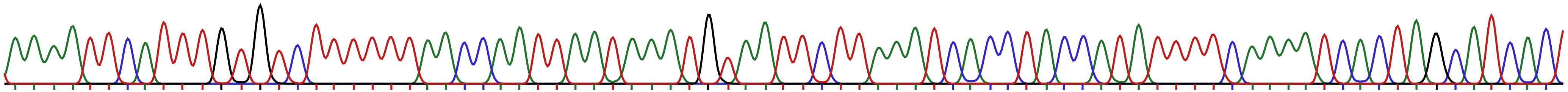

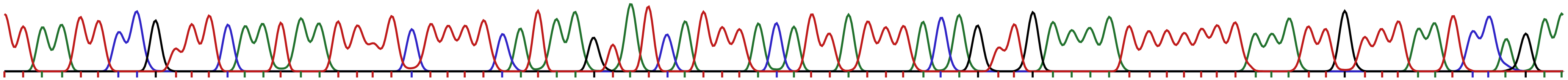

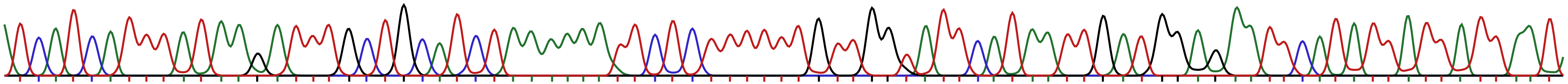

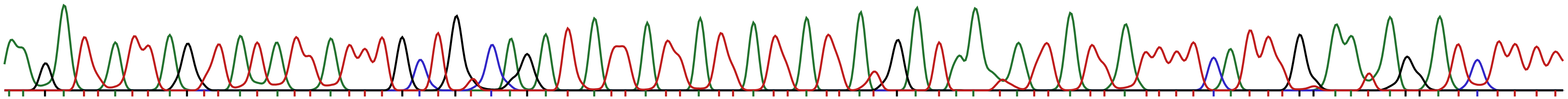

Samples:	12912
Bases:	1095
Average spacing:	12

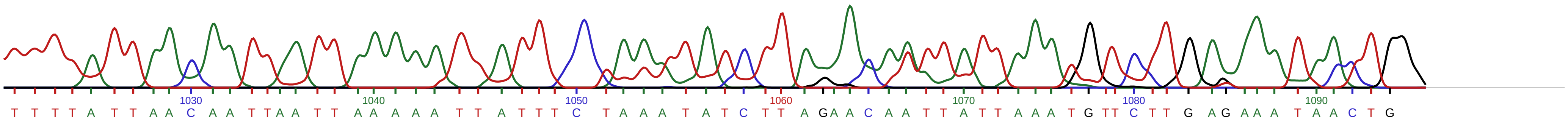

Supplement: Supporting information 2 — (ZIP) [file pone.0316479.s002.zip › 143KN2R_PREMIX_Plate_KELCH2_H04.pdf]
